# The effect of increased weight loading on body weight is partly dependent on Piezo1 in osteoblast-lineage cells and TrkA signaling

**DOI:** 10.1038/s41598-026-40431-8

**Published:** 2026-02-18

**Authors:** Daniel Hägg, Lei Li, Alec T. Beeve, Erica L. Scheller, Jakob Bellman, Fredrik Anesten, Jovana Zlatkovic, Adrià Dalmau Gasull, Ferran Font-Gironès, Sofia Movérare-Skrtic, John-Olov Jansson, Claes Ohlsson

**Affiliations:** 1 Department of Internal Medicine and Clinical Nutrition, Institute of Medicine, Sahlgrenska Osteoporosis Centre, Centre for Bone and Arthritis Research at the Sahlgrenska Academy, University of Gothenburg, Gothenburg, Sweden; 2 Department of Medicine, Division of Bone and Mineral Diseases, Washington University, Saint Louis, MO USA; 3 Department of Biomedical Engineering, Washington University, Saint Louis, MO USA; 4 Department of Physiology, Institute of Neuroscience and Physiology, Sahlgrenska Academy, University of Gothenburg, Gothenburg, 41390 Sweden

**Keywords:** Cell biology, Physiology

## Abstract

Obesity is a complex disease driven by multiple factors, and a deeper understanding of its underlying mechanisms could enable the development of novel treatments. Based on our previous experimental studies, we have proposed a homeostatic mechanism regulating adiposity involving mechano-sensing of body weight by osteoblast-lineage cells in the lower extremities. However, the molecular mechanism underlying this proposed weight-sensing pathway remains to be elucidated. Recent studies have demonstrated that Piezo1-mediated mechano-sensing in osteoblast-lineage cells, as well as TrkA-dependent signaling, are essential for the normal bone anabolic response to high-intensity mechanical loading. We hypothesized that these pathways within bone may also contribute to the sensing of sustained increased weight loading, thereby influencing the homeostatic regulation of body weight. To test this hypothesis, we first established a high-fat diet-induced obesity mouse model with conditional deletion of Piezo1 in osteoblast-lineage cells. Our results demonstrate that the effect of increased weight loading, induced by implanted weights, on body weight reduction is partially dependent on Piezo1 expression in osteoblast lineage cells. Similarly, using a mouse model lacking functional TrkA signaling, we demonstrated that the response to increased weight loading on body weight reduction is partially dependent on functional TrkA signaling. In conclusion, we demonstrate that the effect of increased weight loading on body weight is at least partially dependent on Piezo1 expression in osteoblast-lineage cells and intact TrkA signaling. Based on these findings we propose that increased body weight, resulting from adiposity, may be sensed by osteoblast-lineage cells through Piezo1 activation and that intact TrkA function is necessary for the weight-reducing response to increased weight loading. This mechanosensory input may then initiate compensatory central pathways that reduce adiposity and body weight.

**Supplementary Information:**

The online version contains supplementary material available at 10.1038/s41598-026-40431-8.

## Introduction

Obesity is a chronic disease affecting a substantial proportion of the global population. It is associated with a wide range of comorbidities, including type 2 diabetes, cardiovascular disease and certain cancers^1–3^. Obesity treatment has recently advanced significantly with the introduction of GLP-1 and combined GLP-1 and GIP receptor agonists^4–6^. However, these pharmacological treatments reduce body weight both via a reduction of fat mass and lean mass. The associated loss of lean mass may be problematic in individuals with sarcopenic obesity, a condition characterized by low muscle mass and strength in the context of excess adiposity^7–10^. We recently observed that increased loading through the use of weighted vests reduces fat mass in humans, while lean mass remains unchanged^11^or is slightly increased^12^. Based on these findings, we propose that weight loading may serve as a complementary strategy to GLP-1-based therapies in individuals with sarcopenic obesity—not only to augment fat loss, but also to preserve or potentially increase lean mass.

We have previously proposed a regulatory mechanism of adiposity, involving mechanosensing of increased body weight by osteoblast-lineage cells in the lower extremities^13–17^. However, the molecular mechanism underlying this proposed weight-sensing process remains to be elucidated. Recent studies suggest that both Piezo1-mediated mechano-sensing in osteoblast-lineage cells^18–21^, and NGF-TrkA-dependent signaling^22^, are essential for the normal bone anabolic response to high-intensity mechanical loading. Based on these findings, we hypothesize that these pathways may also contribute to the sensing of sustained increased weight loading, potentially influencing the regulation of adiposity and body weight.

The mechanoreceptor Piezo1, first described in 2010^23^, responds to mechanical force through a mechanism where alterations of membrane shape trigger channel opening which allow for ion transport^24^. Piezo1 is essential for mechanotransduction in various cell types such as red blood cells, skeletal muscle cells and neurons, and activation of Piezo1 triggers cell- and tissue-specific downstream effects^25^. TrkA, the high-affinity receptor for nerve growth factor (NGF), is essential for the development, survival, and functional maintenance of sensory neurons. NGF binding to TrkA initiates a signaling cascade that supports nociceptive transmission^26^.

The aim of the present study was to determine if Piezo1 in osteoblast-lineage cells and/or TrkA-mediated signaling are involved in the response to sustained increased weight loading, induced by implanted weights, on adiposity and body weight regulation.

## Materials and methods

### Animals

All animal research was approved by the regional animal ethics committee in Gothenburg, Sweden, and all experiments were performed in accordance with the ethical approval as well as national and local guidelines and regulations. The report on experimental animal utilization is to the best of our effort reported in accordance with the ARRIVE guidelines. The animals were kept in a standard animal facility under controlled temperature (22 °C) with 12 h light/dark cycle, with free access to fresh water and ad libitum normal chow (Harlan, NJ, USA). Prior to loading experiments, the animals were fed a high-fat diet (HFD, D12892; Research Diets, New Brunswick, NY, USA) for six to eight weeks. All mice used in this project were on C57BL/6 background. Wild-type mice type was purchased from Jackson Laboratory (C57BL/6J, #000664).

To specifically inactivate *Piezo1* in the osteoblast-lineage, *Piezo1*^flox/flox^ mice (Jackson Laboratory, strain #029213)^27^were crossed with *Runx2*-Cre mice (kindly provided by Professor Jan Tuckermann, Ulm University, Germany), which have been previously shown to express Cre recombinase in early osteoblast-lineage cells with an unchanged skeletal phenotype compared to WT mice^28^.

To generate mice with Piezo1 depletion in osteoblasts (denoted *Runx2*-Cre;*Piezo1*^flox/flox^), *Piezo1*^flox/wt^ mice were bred with littermates carrying one *Runx2*-Cre allele (*Runx2*-Cre;*Piezo1*^flox/wt^). As male controls, *Piezo1*^flox/flox^ mice lacking the Runx2-Cre allele were used. The offspring were not in mendelian distribution, with relatively small litter sizes. For the female mice, experiments were performed using mice from two breeding rounds. One compared *Piezo1* ^flox/flox^ with *Runx2*-Cre;*Piezo1*^flox/flox^ and one compared *Piezo1* ^flox/flox^ with heterozygous *Piezo1*^flox/wt^ mice carrying the *Runx2*-Cre allele (denoted *Runx2*-Cre;*Piezo1*^flox/wt^). There was no difference in response to increased loading between *Piezo1*^flox/flox^ mice and *Runx2*-Cre;*Piezo1*^flox/wt^; supplementary Fig. 1a and b. Therefore, to achieve adequate statistical power in the female loading experiment, these two groups were pooled together (Fig. 2c, denoted *Piezo1* ^flox/flox^ + *Runx2*-Cre;*Piezo1*^flox/wt^ ) and used as controls compared to the *Runx2*-Cre;*Piezo1*^flox/flox^ mice (Fig. 2d).

*TrkA* ^*F592A*^ mice (Jackson Laboratory, strain #022362) were originally developed by Professor David Ginty at Johns Hopkins University^29^and they carry a phenylalanine to alanine (F592A) point mutation in exon 12 of the *TrkA* gene. This point mutation allows for the complete blocking of TrkA signaling upon administration of the small molecule 1NM-PP1. The day before the weight loading experiment started, 1NM-PP1 (MedKoo, #406395, 40 µM in ddH2O supplemented with 1% PBS-Tween-20) was added to the drinking water of all mice included in the experiment. Water was changed twice a week and 1NM-PP1 treatment continued throughout the duration of the experimental period, as described by Tomlinson et al.^22^. Breeding was performed by mating heterozygous mice. Mice that were homozygous for the mutation were referred to as *TrkA*^*F592A*^, and wild-type littermates were used as controls, referred to as *TrkA*^*WT*^.

### Weight loading surgery

Loading of mice fed a high-fat diet was performed as described previously^13^. Due to breeding latency, animals were between eight and sixteen weeks old when they were put on high-fat diet. Prior to surgery, the animals were weight-matched into control or experimental groups. Starting weights for *Piezo1*^flox/flox^ mice were 40.5 ± 1.7 g for female control group, 40.9 ± 1.4 g for female load group, 44.7 ± 1.5 for male control group and 45.5 ± 1.1 g for male load group. Starting weights for *Runx2*-Cre;*Piezo1*^flox/flox^ female control group was 35.2 ± 4.4 g, 34.1 ± 2.7 g for female load group, 34.8 ± 1.9 g for male control group and 34.2 ± 1.9 g for male load group. Starting weights for *TrkA*^WT^ were 38.2 ± 1.3 g for female control group, 38.1 ± 1.1 g for female load group, 46.9 ± 1.7 g male control group and 47.6 ± 1.5 g for male load group. For *TrkA*^F592A^ the starting weights were 32.9 ± 2.1 g for female control group, 35.0 ± 2.8 g for female load group, 42.9 ± 2.0 g for male control group and 40.9 ± 2.0 g for male load group. The animals were sedated using isoflurane. Anesthesia was induced in a small container at 3–5% volume in air. After the animal was asleep, it was transferred to the operating table with a mask covering its snot, and anesthesia was maintained at 2–3%. Under isoflurane anesthesia, the abdominal skin was shaved and disinfected with chlorhexidine. A midline incision was made, and the peritoneum was opened. A cylindrical capsule made of sustarin C (Röchling, Mannheim, Germany) was inserted in the abdominal cavity. In the group with increased load (referred to as load), the capsule was filled with tungsten powder (Edstraco AB, Rönninge, Sweden) corresponding to 15% of the animal’s body weight. In the control group, an empty capsule, weighing 2–3% of the animal’s body weight was inserted. Peritoneum was closed using 5 − 0 nylon suture (Medtronic, Galway, Ireland) and the skin was closed using agraff (AgnTho’s, Solna, Sweden). Analgesia was given prior to surgery and again 24 h postoperatively. Animals were weighed several times a week (as specified for each experiment) over a two-week period before the experiment ended. After body weight was recorded, the weight of the capsule was subtracted, to calculate percent change in body weight from baseline. Animal welfare was examined daily for signs of discomfort or severe pain. This included lethargic behavior (not moving), piloerection, poor wound healing, excessive weight loss, low body temperature (cold when touched). Animals in poor condition were euthanized. A total of 182 loading surgeries were performed, and 19 animals (19/182 = 10%) were excluded. 163 animals were included, 84 males and 79 females.

At experimental endpoint, the animals were euthanized, and retroperitoneal white adipose tissue was dissected out and weighed. Euthanasia was performed by cervical dislocation after isoflurane anesthesia. The animals were sedated using isoflurane. Anesthesia was induced in a small container at 3–5% volume in air.

### Quantitative real-time PCR analyses of Piezo1 expression in bone

After euthanasia, tibiae were dissected and surrounding tissues were carefully removed. The bones were immediately transferred to RNAprotect Tissue reagent (Qiagen, Hilden, Germany) and stored in -70 °C. Total RNA were extracted from cortical bone (tibial shafts flushed to remove bone marrow) using TRIzolReagent (#15596018, Thermo Fisher Scientific, Waltham, MA), followed by purification using the RNeasy Mini Kit (#74116, Qiagen). cDNA was synthesized using the High-Capacity cDNA Reverse Transcription Kit (4368814, Thermo Fisher Scientific). Gene expression analysis was performed on StepOne Plus Real-Time PCR System (Thermo Fisher Scientific) using the TaqMan assay Mm01241547_g1 for Piezo1 and using 18 S (4310893E) as endogenous control. Relative expression levels were calculated using the 2^−ΔΔCt^ method.

### Peripheral quantitative computed tomography (pQCT)

After euthanasia, tibiae were dissected and fixed in 4% paraformaldehyde for 48 h before transfer to 70% ethanol. Peripheral quantitative computed tomography (pQCT; XCT Research M, Stratec Medizintechnik GmbH, Germany) was used to analyze the cortical compartments of the tibia at a resolution of 70 μm. Briefly, the cortical bone was analyzed in the mid-diaphyseal region at 30% of the total length of the bone from the proximal growth.

### RNA scope in situ hybridization, image analysis, and quantification

After euthanasia, tibiae were dissected and fixed in 4% paraformaldehyde for 48 h before transfer to 70% ethanol. The tibiae were embedded in paraffin and sectioned in 6 μm thick Sects.  2–3 slices were used from each animal. RNAscope-based in situ hybridization was performed to visualize *Piezo1* and *Runx2* transcript localization. Chromogenic detection was carried out using the RNAscope Duplex Detection Kit (Advanced Cell Diagnostics (ACD), 322500) following the manufacturer’s instructions. Specific probes targeting *Piezo1* (channel C1; ACD; 529091) and *Runx2* (channel C2; ACD, 414021-C2) were used. Chromogenic signals were developed using the kit’s green chromogen for channel C1and fast red for channel C2. Counterstaining was performed using 50% hematoxylin. Whole-slide imaging was performed using a Zeiss slide scanner.

Quantitative analysis of chromogenic RNAscope-stained tibial cortical bone section was performed to assess the efficiency of *Piezo1* deletion in *Runx2*-expressing cells. Within defined regions of interest, osteocytes were manually evaluated for the presence of *Piezo1* and/or *Runx2* transcripts. The proportion of *Piezo1*-positive cells among total osteocytes was calculated, and the percentage of *Runx2*-positive cells co-expressing *Piezo1* was used as a measure of Cre-mediated knockout efficiency. To further determine whether *Piezo1* expression persisted in non-*Runx2*-expressing cell populations, adjacent skeletal muscle tissue was analyzed. Due to limitations in delineating individual myocytes after hematoxylin counterstaining, *Piezo1* expression in muscle was quantified as transcript density (puncta per mm²), using standardized thresholding in Fiji (ImageJ).

### Immunostaining for calcitonin gene-related peptide (CGRP) and tyrosine hydroxylase (TH)

Left tibiae of male wild type control and loaded C57Bl6/J mice were collected and processed for fixed frozen histology and nerve immunostaining as described previously^30,31^. Briefly, tissues were embedded and sectioned at 50 µm using a cryostat (Leica, Buffalo Grove, IL, USA) onto Colorfrost Plus glass slides (Thermo Fisher Scientific 12-550‐18). The sections were blocked in 10% donkey serum in TNT buffer, followed by incubation for 48-hours at 4°C using primary antibodies (anti-Calcitonin Gene Related Peptide, Bio-Rad, 1720–9007, 1:1000; anti-Tyrosine Hydroxylase, Abcam, ab152, 1:1000). After washing, sections were incubated with secondary antibodies (1:500 ) in TNT buffer for 24 hours at 4°C, washed and counterstained using DAPI (Sigma‐Aldrich) for 5 minutes, thereafter washed and mounted using Fluoromount‐G (Thermo Fisher Scientific 00‐4958‐02). Serial tiled images were acquired using a spinning disk confocal microscope Nikon (Tokyo, Japan) with a 10× objective (pixel size = 0.650 µm, step size = 2.5 µm, number of steps = 21). Image stitching of nd2 multipoint files was done using the FIJI Stitching plugin (“snake by rows” Mode). Nerve axon density was quantified based on 50 µm thick sections using standardized protocols and morphologic criteria as previously reported^30,32^. Detailed step-by-step protocols are also available for nerve immunostaining/imaging (https://www.protocols.io/view/spatial-mapping-and-contextualization-of-axon-subt-36wgq4nnkvk5/v1) and nerve tracing (https://www.protocols.io/view/protocol-for-quantification-of-bone-indices-calcei-bp2l62b1dgqe/v1).”

### Tail-flick experiment

Peripheral nerve sensitization of the *TrkA*^*F592A*^ mice was analyzed using the tail-flick method^33^. To induce TrkA inhibition, 40 µM 1NM-PP1 was added to the drinking water the day before experiment start. On day one, the tip of each mouse’s tail was dipped in room temperature water for five seconds, then dipped in 52 °C water. The time from submersion to reaction (lifting of the tail) was recorded as reaction time. Immediately after the experiment, the mice received an intraperitoneal injection of the TrkA agonist gambogic amide (Alomone Lab #G-235), 4 mg/kg to induce peripheral sensitization^34^. The next day, the procedure was repeated, and the change in reaction time was recorded.

### Statistics

Statistical analyses were performed using GraphPad prism version 10. (GraphPad Software, MA, USA). Repeated-measures ANOVA was used to analyze the overall effect of increased loading on body weight change. Statistical analyses were based on body weight measurements collected at each time point during the experiment. A sex-combined linear regression analysis of body weight change at the end of the study, adjusting for sex and including an interaction term between genotype and loading procedure (genotype * loading procedure) was used to determine if the loading response differed between genotypes. For comparison between two groups, unpaired Student’s t-test (RT -PCR, RNA Scope, neurite length and number) or paired Student’s t-test (tail-flick) was used. Results are presented as mean values ± SEM. A P-value < 0.05 was considered statistically significant.

## Results

### The effect of increased weight loading on body weight is partly dependent on Piezo1 in Runx2 expressing osteoblast-lineage cells

**Fig. 1:**
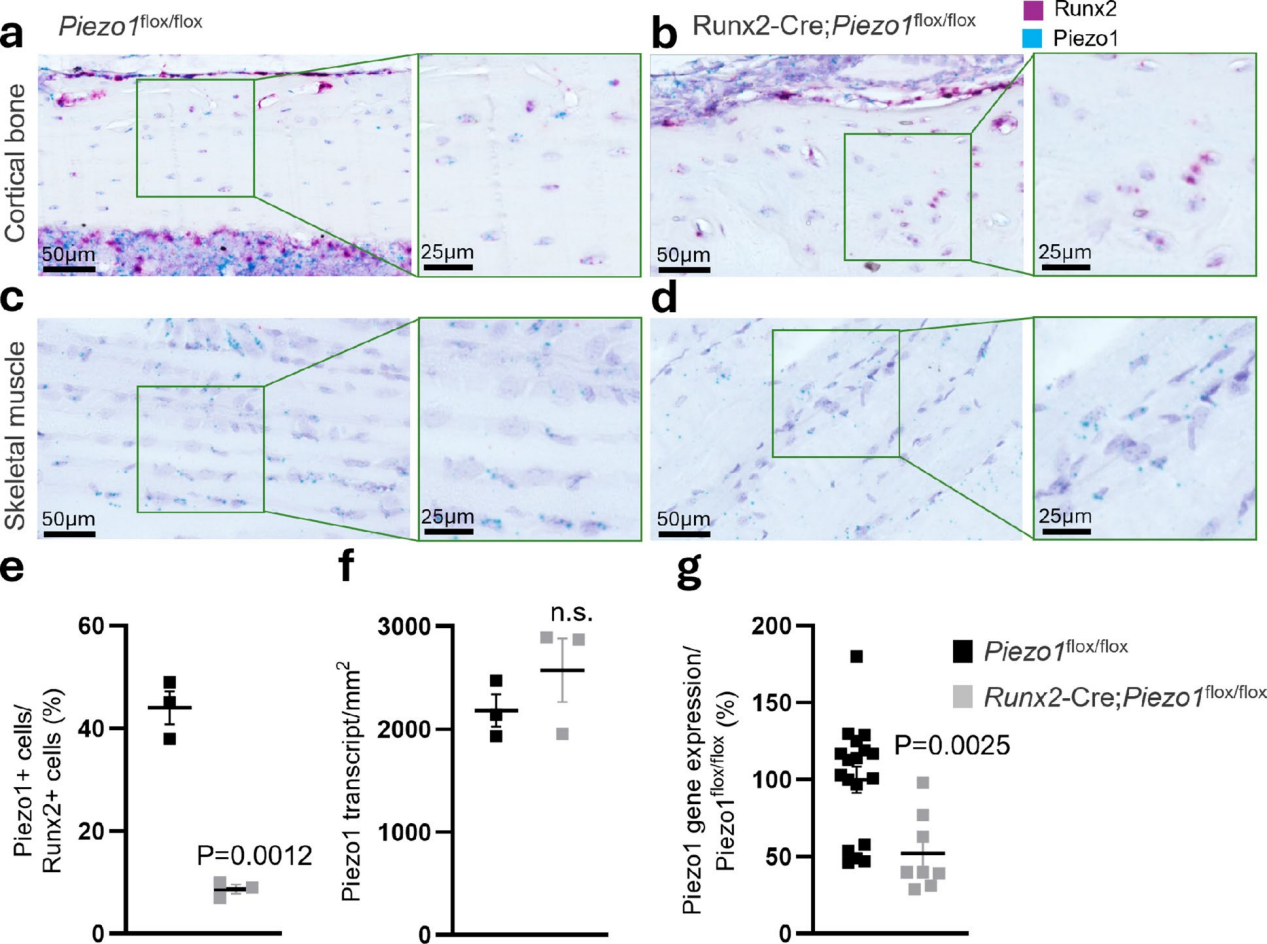
Specific deletion of Piezo1 in Runx2 expressing osteoblast-lineage cells. (**a**) RNA scope chromogenic in situ hybridization confirmed *Piezo1* (cyan) expression in osteoblast-lineage cells (*Runx2*; red) in cortical bone of tibia in male mice (*Piezo1*^flox/flox^). (**b**) Specific deletion of *Piezo1* in Runx2-positive cells in cortical bone (*n* = 3 per group). (**c**, **d**) RNA scope chromogenic in situ hybridization of adjacent skeletal muscle of *Piezo1*^flox/flox^ and *Runx2*-Cre;*Piezo1*^flox/flox^ male mice respectively. (**e**) The number of Runx2-positive cells also expressing *Piezo1* was significantly reduced in *Runx2*-Cre;*Piezo1*^flox/flox^ mice compared to *Piezo1*^flox/flox^ mice. (**f**) *Piezo1* transcript density in adjacent skeletal muscle, quantified by automated puncta counting and normalized to tissue area, was unaffected in *Runx2*-Cre;*Piezo1*^flox/flox^ mice compared to *Piezo1*^flox/flox^ mice (*n* = 3 per group). (**g**) Piezo1 mRNA expression analysis of tibia cortical bone (*Piezo*^*flox/flox*^ male mice *n* = 18, *Runx2*-Cre;*Piezo1*^flox/flox^ male mice *n* = 8). Expression of *Piezo1* mRNA is given as % of *Piezo*^*flox/flox*^). Data are shown as mean ± SEM. Statistical significance was determined using Student’s t-test; *p* < 0.05 was considered significant.

To determine whether Piezo1 in osteoblast-lineage cells contributes to regulation of body weight in response to sustained increased weight loading, we established a high-fat diet-induced obesity mouse model with deletion of Piezo1 in *Runx2*-expressing osteoblast-lineage cells (denoted *Runx2*-Cre;*Piezo1*^*flox/flox*^). Expression of Piezo1 in osteoblast-lineage cells of wild-type mice (*Piezo1*^flox/flox^) was confirmed using RNA scope chromogenic in situ hybridization of tibia sections (Fig. 1a). Specific deletion of Piezo1 in *Runx2*-Cre;*Piezo1*^*flox/flox*^ mice was supported by an 80% reduction in the number of *Runx2*-positive osteoblast-lineage cells co-expressing *Piezo1* in cortical bone (Fig. 1b and c) while *Piezo1* transcript density in adjacent skeletal muscle remained unchanged in *Runx2*-Cre;*Piezo1*^*flox/flox*^ mice compared with *Piezo1*^flox/flox^ mice (Fig. 1d). Real-time-PCR analyses of tibia cortical bone confirmed a down regulation of *Piezo1* mRNA expression levels in *Runx2*-Cre;*Piezo1*^*flox/flox*^ mice compared with *Piezo1*^flox/flox^ control mice (Fig. 1e).

**Fig. 2:**
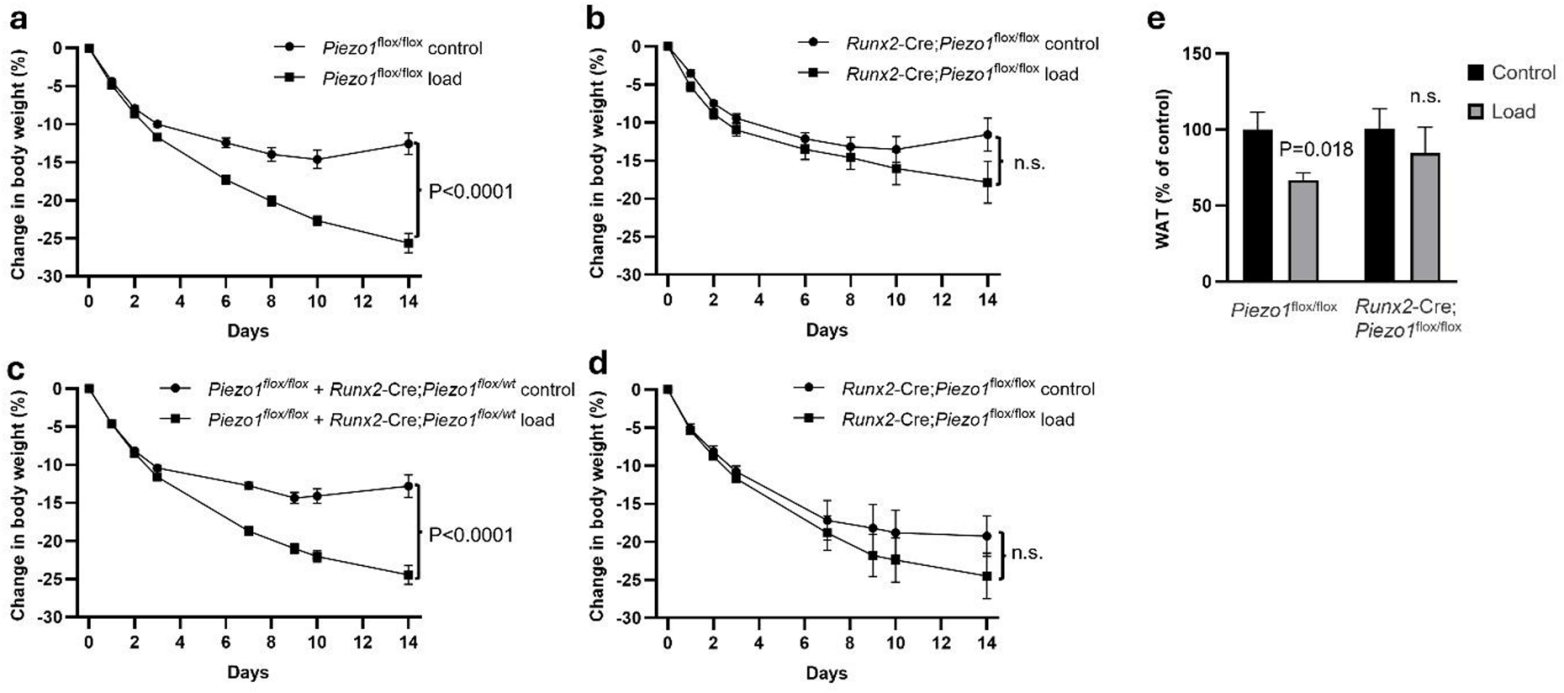
Piezo1 in Runx2-expressing osteoblast-lineage cells partly mediates the effect of increased weight loading on body weight. (**a**–**d**) The effect of increased loading on body weight in mice with either intact *Piezo1* expression (*Piezo1*^flox/flox^) or reduced *Piezo1* expression in *Runx2*-expressing osteoblast-lineage cells (*Runx2*-Cre;*Piezo1*^flox/flox^). (**a**) Male *Piezo1*^flox/flox^ mice (control *n* = 15, load *n* = 13), (**b**) male *Runx2*-Cre;*Piezo1*^flox/flox^ mice (control *n* = 9, load *n* = 9), (**c**) female *Piezo1* ^flox/flox^ + Runx2-Cre; Piezo1^flox/wt^ mice (control *n* = 19, load *n* = 17), and (**d**) female *Runx2*-Cre;*Piezo1*^flox/flox^ mice (control *n* = 5, load *n* = 8). (**e**) The effect of increased loading on retroperitoneal white adipose tissue (WAT) weight in male *Piezo1*^flox/flox^ (control *n* = 15, load *n* = 13) and *Runx2*-Cre;*Piezo1*^flox/flox^ (control *n* = 9, load *n* = 9) mice. Data are shown as mean ± SEM. Statistical analyses were performed using repeated measures ANOVA (for weight curves) or Student’s t-test (for retroperitoneal WAT). *p* < 0.05 was considered significant., n.s = non-significant.

Mice were treated with either an implanted empty capsule (control) or a capsule weighing 15% of their body weight (load). Male mice with intact Piezo1 expression (*Piezo1*^*flox/flox*^) demonstrated a significant reduction in body weight in response to increased weight loading (Fig. 2a, Figs. S2a, S3a), whereas in male mice with reduced *Piezo1* expression in *RunX2* expressing osteoblast-lineage cells (*Runx2*-Cre;*Piezo1*^*flox/flox*^) there was no significant reduction in body weight between load and control groups (Fig. 2b, Figs. S2b, S3b). Similar findings were observed in female mice (Fig. 2c and d, Figs. S2c and d, S3c and d). A sex-combined linear regression analysis of body weight change at the end of the study, adjusted for sex, showed a significant interaction term between genotype and loading (*p* = 0.040). This finding demonstrates that the body-weight reduction in response to increased weight loading was attenuated in mice with deletion of *Piezo1* in *Runx2* expressing osteoblast-lineage cells.

Increased weight loading reduced the weight of retroperitoneal white adipose tissue (WAT) in male *Piezo1*^*flox/flox*^ mice by- 37% but no such effect was observed in *Runx2*-Cre;*Piezo1*^*flox/flox*^ mice (Fig. 2e). Although *Runx2*-Cre;*Piezo1*^flox/flox^ mice exhibited reduced cortical cross-sectional area, cortical thickness and bone length in tibia compared to *Piezo1*^flox/flox^ mice, increased weight loading did not affect these bone parameters in either genotype (Fig S1c–e).”

### The effect of increased weight loading on body weight is partly dependent on TrkA mediated signaling

TrkA signaling is enriched in several neural populations and can control nerve growth and function^35,36^. TrkA signaling has been described as participating in regulation of bone homeostasis and response to mechanical load^22,37^. We hypothesized that TrkA signaling, potentially through local nerves, could interact with the role of Piezo1 in bone to mediate the response to increased weight loading. As is well-established^38^, we observed that sensory nerves (CGRP positive axons) and a smaller number of sympathetic nerves (TH positive axons) are abundant in the tibia (Fig. S4a and b). In a descriptive analysis, we first confirmed previous studies that sensory nerves (CGRP positive axons) and a smaller number of sympathetic nerves (TH positive axons) are present in the tibia (Fig. S4a and b). A previous study showed that high intensity mechanical loading of ulnae activated nerve sprouting locally in the bone^22^. However, in the present study sustained increased weight loading of tibia for 14 days in wild type mice did not increase neurite length density (Fig. S4c) or neurite number (Fig. S4d) when evaluated in the periosteum.

We next examined if the effect of increased loading was dependent on functional TrkA signaling using the inducible global inhibition mouse model TrkA^F592A^. 1NM-PP1 blocks TrkA phosphorylation and activation and thereby inhibits NGF signaling in TrkA^F592A^ but not TrkA^WT^ mice^29^. To confirm TrkA blocking after supplement of 1NM-PP1, a tail-flick experiment was performed. The tail of each mouse was dipped in 52 °C water before the NGF analogue gambogic amide was injected, and the experiment was repeated 24 h later. TrkA^WT^ mice, but not TrkA^F592A^ mice, developed hyperalgesia and their response to heat was quicker at the second measurement (Fig. S1f). This finding demonstrates that TrkA signaling in TrkA^F592A^ mice, but not in TrkA^WT^ mice, can be blocked by adding 1NM-PP1.

**Fig. 3:**
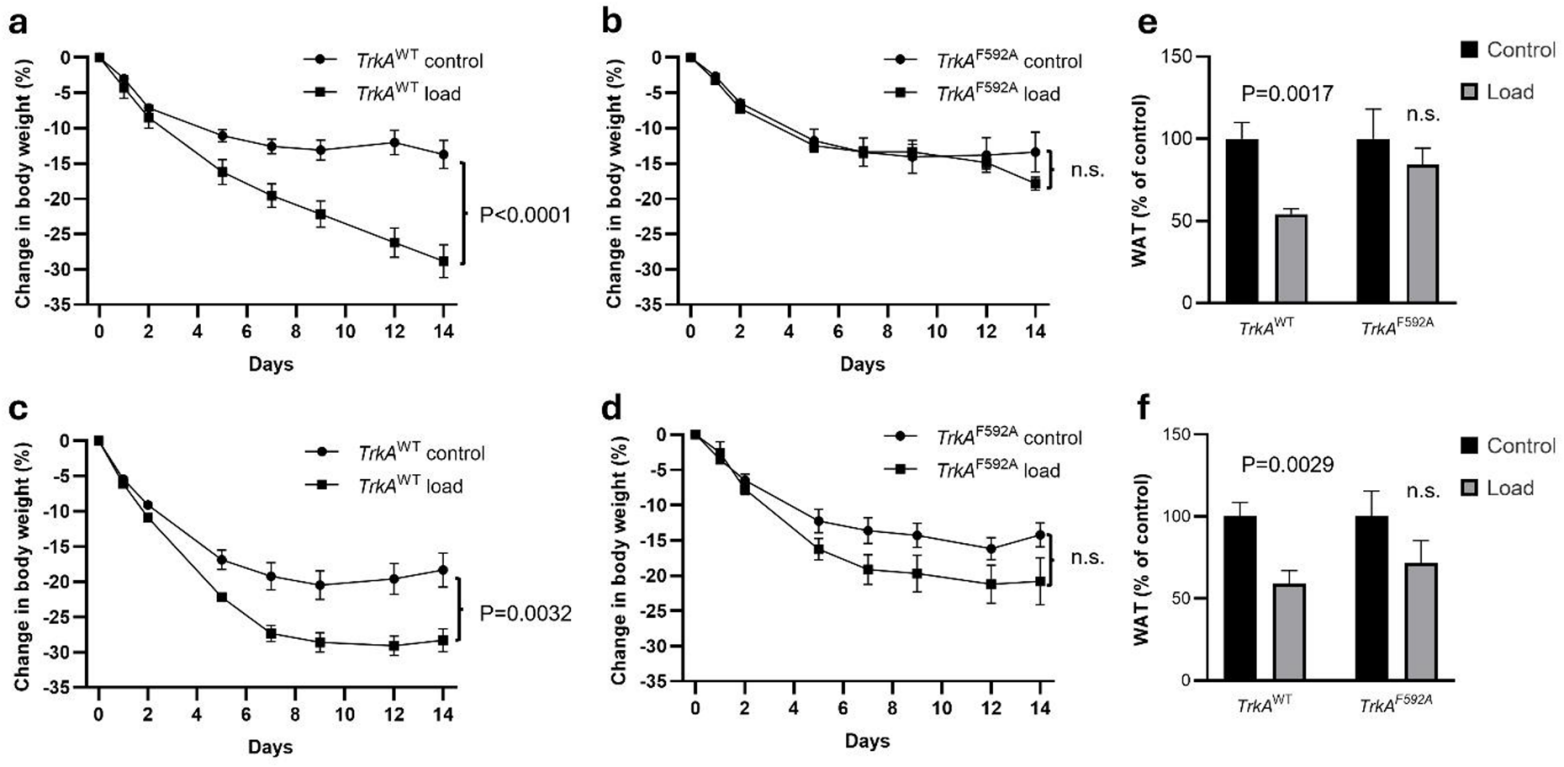
The effect of increased weight loading on body weight is partly dependent on TrkA mediated signaling. (**a**–**d**) The effect of increased loading on body weight in mice with either intact peripheral nerve signaling (*TrkA*^*WT*^) or mice lacking functional TrkA signaling (*TrkA*^*F592A*^). (**a**) Male *TrkA*^*WT*^ mice (control *n* = 7, load *n* = 7), (**b**) male *TrkA*^*F592A*^ mice (control *n* = 7, load *n* = 6), (**c**) female *TrkA*^*WT*^ mice (control *n* = 8, load *n* = 10), and (**d**) female *TrkA*^*F592A*^ mice (control *n* = 7, load *n* = 5). (**e**, **f**) The effect of increased weight loading on retroperitoneal white adipose tissue (WAT) weight in male (**e**) and female (**f**) mice. Data are shown as mean ± SEM. Statistical analyses were performed using repeated measures ANOVA (for weight curves) or Student’s t-test (for retroperitoneal WAT). *p* < 0.05 was considered significant., n.s = non-significant.

Both male (Fig. 3a, Figs. S5a, S6a) and female (Figs. 4c, S5c, S6c) *TrkA*^*WT*^ mice with intact peripheral nerve signaling exhibited significant body weight loss in response to increased weight loading. In contrast, this response was attenuated and not statistically significant in either male (Fig. 3b, Figs. S5b, S6b) or female (Fig. 3d, Figs. S5d, S6d) TrkA^F592A^ mice. A sex-combined linear regression analysis of body weight change at the end of the study, adjusting for sex, showed an overall significant interaction term (*p* = 0.036) between genotype and loading. This finding demonstrates that the response to increased loading on body weight was reduced in TrkA^F592A^ mice.

Increased weight loading also reduced the weight of retroperitoneal WAT in TrkA^WT^ mice while this reduction did not reach statistical significance in TrkA^F592A^ mice (Fig. 3e and f).

## Discussion

Obesity is a chronic condition associated with several serious diseases. Although GLP-1 and combined GLP-1 and GIP receptor agonists have significantly improved obesity treatment, their associated reduction of lean mass may pose risks for individuals with sarcopenic obesity^7–10^. This highlights a medical need for novel treatments selectively reducing fat mass while maintaining lean mass. We recently demonstrated that increased weight loading using weighted vests reduces fat mass without reducing lean mass in humans^11,12^, suggesting that it might be beneficial to combine GLP1 receptor agonists with treatment with weighted vests. Our previous experimental studies in rodents suggest that osteoblast-lineage cells in the lower extremities sense increased body weight due to adiposity through a mechanosensory mechanism (denoted the gravitostat)^13–17^. However, the molecular basis of this proposed weight-sensing pathway has remained unclear. We, herein, used two different gene-targeted mouse models to demonstrate that the effect of increased weight loading on adiposity and body weight is at least partly dependent on Piezo1 expression in osteoblast-lineage cells and intact TrkA signaling.

Bone responds to high intensity loading by increasing local bone formation, a process that includes a mechano-sensing pathway. This anabolic response involves Piezo1-mediated mechano-sensing in osteoblast-lineage cells^18–21^as well as global TrkA-dependent signaling^22^. We investigated whether these pathways may also contribute to the sensing of sustained increased weight loading, which could potentially influence the homeostatic regulation of body weight. Our findings indicate that the effect on body weight by sustained increased weight loading is at least partly dependent on Piezo1 in osteoblast-lineage cells and TrkA signaling. A key distinction between the bone anabolic response to high-intensity mechanical loading and the response to sustained increased weight loading is that the former elicits a localized effect in bone, whereas the latter induces a systemic response on adiposity and body weight regulation. The downstream signaling elicited by sustained increased weight loading likely involves a bone-derived signal that communicates with central regulatory centers governing energy balance and body weight homeostasis^17^.

Local peripheral nerves in the bone modulate but are not required for skeletal adaptation to applied load in mice^39–42^. The NGF receptor TrkA is widely expressed in peripheral sensory and sympathetic neurons^43^, including within the bone-lining periosteum^35^. Global inhibition of TrkA signaling decreases the anabolic response to biomechanical load^22^. This may be through inhibition of TrkA signaling in the peripheral nervous system, or through other mechanisms that remain undefined. One study showed that high intensity mechanical loading induces local nerve sprouting near the ulna bone^22^. By contrast, a recent report using a comparable bone loading model did not observe periosteal nerve sprouting in the tibia despite a pronounced osteoanabolic response to load^32^. Similarly, the sustained increased weight loading in the present study did not induce periosteal sprouting of either CGRP-positive sensory nerves or TH-positive sympathetics in the mouse tibia.

Based on the findings in the present study, we propose that increased weight loading is sensed by osteoblast lineage cells via Piezo1, and that intact TrkA function is necessary for the weight-reducing response to increased weight loading. Future studies are required to determine if the Piezo1 effect is mediated via downstream TrkA activation. We recently observed that the body weight reduction by increased weight loading is associated with sensory signaling in the dorsal horn of the lumbar spinal cord^44^ and activation of neurons in the medial nucleus of the solitary tract^17^. These observations suggest that these two sites may be involved in the downstream signaling of the mechanosensing pathway, ultimately impacting fat mass regulation.

One potential limitation of employing implanted capsules for weight loading in rodent models is the possible induction of a physiological stress response, which may confound experimental outcomes. However, in our previous experimental studies, we have not observed any effect on locomotion^13,14^, markers of stress^14^or inflammation^16^of this weight loading procedure.

In this study, we selected the Runx2 promoter for Piezo1 inactivation because Runx2 is a marker of early osteoblasts and is expected to induce a broad effect by targeting all cells within the osteoblast lineage. This approach was intended to maximize the likelihood of detecting the functional impact of Piezo1 in osteoblast-lineage cells within our experimental model. A limitation of our model employing non-inducible *Runx2*-Cre is the likely occurrence of Piezo1 deletion in chondrocytes^18^, potentially leading to developmental effects prior to the initiation of the study. This is reflected by the lower body weight and reduced bone lengths observed in *Runx2*-Cre mice compared with control mice in the present study.

Using RNAscope, we observed an 80% reduction in *Runx2*-positive cells expressing *Piezo1*; however, real-time PCR analysis of whole diaphyseal cortical bone from the tibia revealed only a 50% decrease in Piezo1 gene expression. The residual Piezo1 expression in the diaphyseal cortical bone of conditional knockdown mice likely originates from non-osteoblast lineage cells lacking *Runx2* expression. Although we provide RNAscope evidence demonstrating reduced Piezo1 mRNA expression and reproduce the previously reported bone phenotype of the same *Runx2*-Cre;*Piezo1*^flox/flox^ mouse model^18^, it is a limitation of the present study that we do not demonstrate Piezo1 knock-down at the protein level.

In conclusion, we demonstrate that the effect of increased weight loading on body weight is dependent on Piezo1 expression in Runx2-positive osteoblast-lineage cells and intact TrkA signaling. Based on these findings we propose a model in which increased weight loading is sensed by osteoblast-lineage cells via Piezo1 activation, and that intact TrkA function is necessary for the weight-reducing response to increased weight loading.

## Supplementary Information

Below is the link to the electronic supplementary material.

Supplementary Material 1

## Data Availability

The datasets generated and analyzed in this study are available upon request from the corresponding author.
