# Supplementary material for: The effect of increased weight loading on body weight is partly dependent on Piezo1 in osteoblast-lineage cells and TrkA signaling
Source: Sci Rep. 2026 Feb 18;16:7162. doi: 10.1038/s41598-026-40431-8 (PMC12920712; doi:10.1038/s41598-026-40431-8)
Supplement: Supplementary file 1 — Supplementary Material 1 [file 41598_2026_40431_MOESM1_ESM.docx]

**
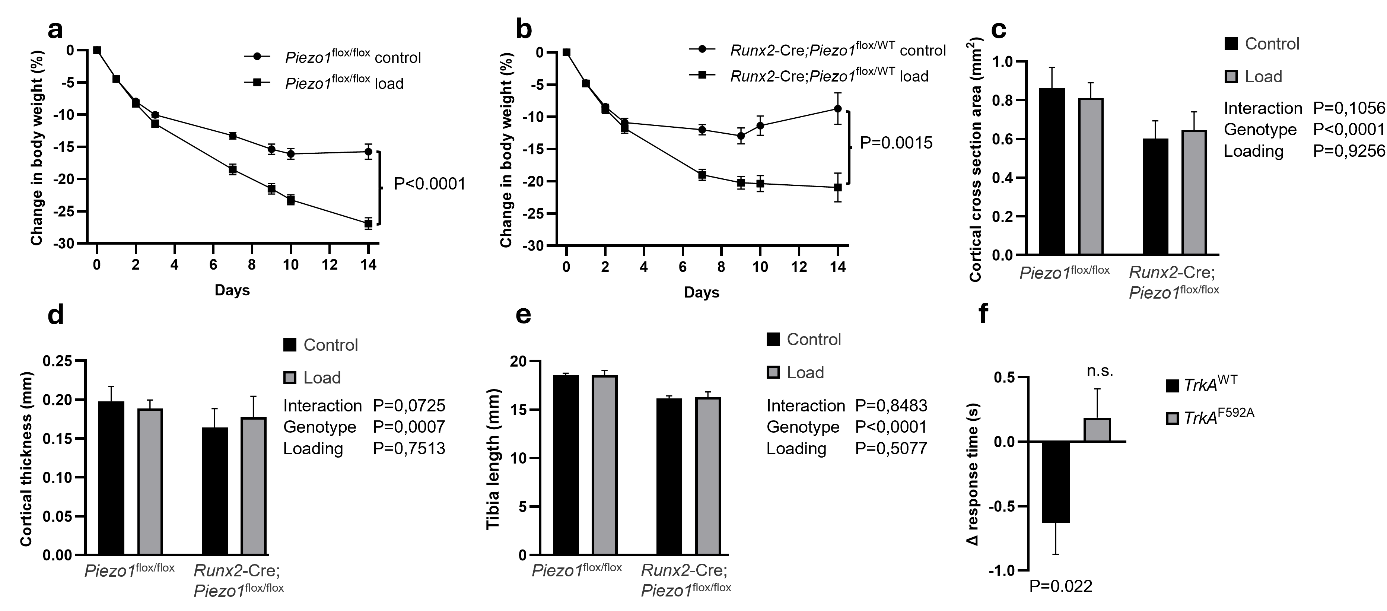
**

**Supplementary figure 1.** For female mice, there was no difference in response to increased loading between *Piezo1*^flox/flox^ mice (**A**, control *n* = 11, load *n* =10) and heterozygous knock-down mice (*Runx2*-Cre;*Piezo1^wt/flox^* mice; **B**, control *n* = 8, load *n* =7).

**(C-E**) Although *Runx2-*Cre;*Piezo1^flox/flox^* mice exhibited reduced cortical cross-sectional area, cortical thickness and bone length in tibia compared to *Piezo1^flox/flox^* mice, increased weight loading did not affect these bone parameters in either genotype (*Piezo1^flox/flox^,* control *n* = 15, load *n* = 13; Runx2-Cre;*Piezo1^flox/flox^* control *n* = 9, load *n* = 9).

(**F**) Tail-flick analysis before and 24 h after the mice were given the TrkA agonist gambogic amide show that TrkA^WT^ mice were sensitized by the treatment and responded faster the second time, whereas TrkA^F592A^ mice did not respond faster after treatment with the NGF analogue (TrkA^WT^, *n* = 15; TrkA^F592A^ n *=* 14).

Data are shown as mean ± SEM. Statistical analyses were performed using repeated measures ANOVA (for weight curves), two-way ANOVA (for bone parameters), or paired Student’s t test (for tail-flick). A p-value <0.05 was considered statistically significant. n.s = non-significant.


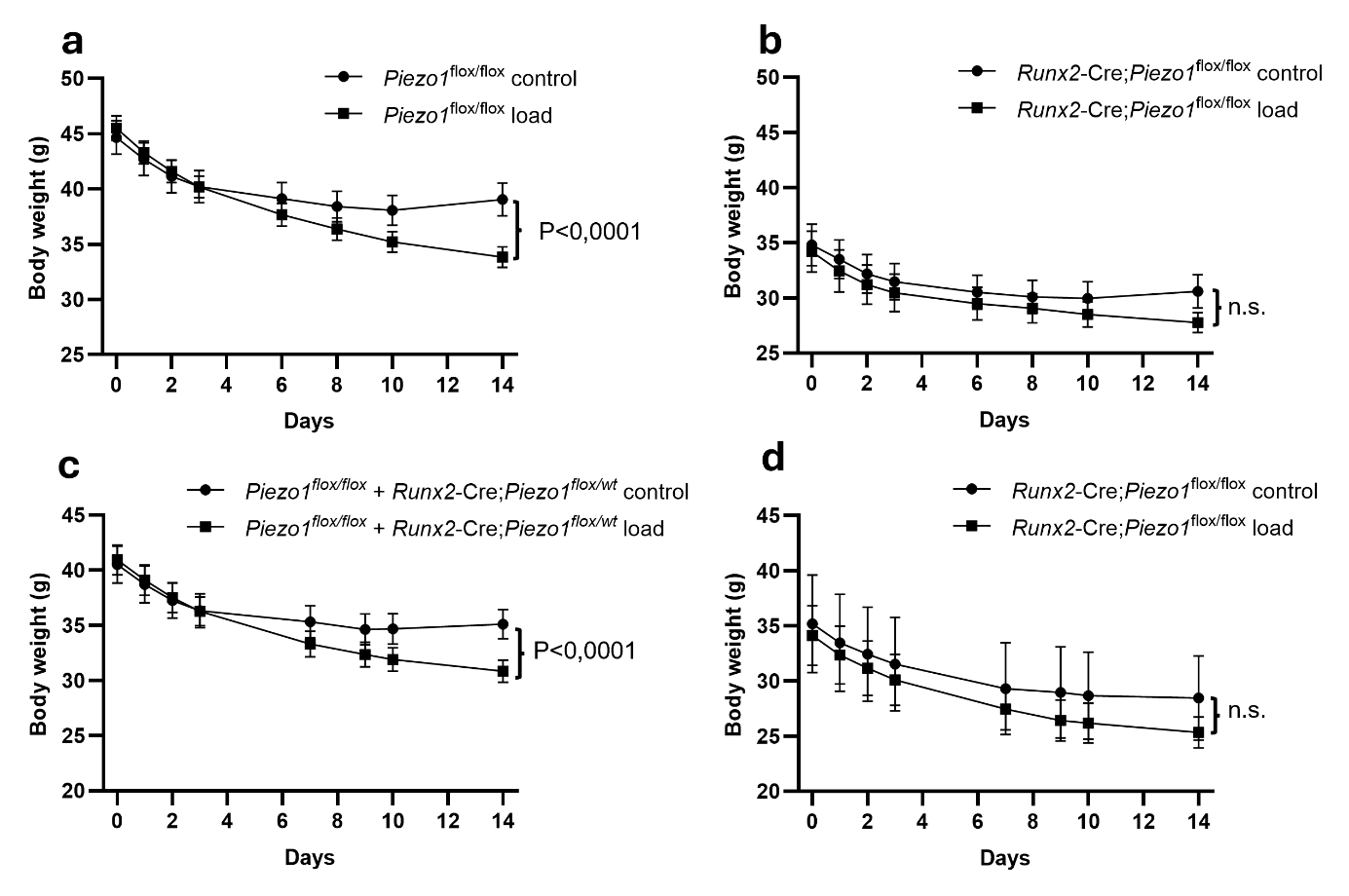


**Supplementary figure 2. Piezo1 in Runx2-expressing osteoblast-lineage cells partly mediates the effect of increased weight loading on body weight**

(A–D) The effect of increased loading on body weight presented as total body weight in mice with either intact *Piezo1* expression (Piezo1^flox/flox^) or reduced *Piezo1* expression in *Runx2*-expressing osteoblast-lineage cells (*Runx2*-Cre;*Piezo1*^flox/flox^). (A) Male Piezo1^flox/flox^ mice (control *n* = 15, load *n* = 13), (B) male *Runx2*-Cre;*Piezo1*^flox/flox^ mice (control *n* = 9, load *n* = 9), (C) female Piezo1 ^flox/flox^ + Runx2-Cre;Piezo1^flox/wt^ mice (control *n* = 19, load *n* = 17), and (D) female *Runx2*-Cre;*Piezo1*^flox/flox^ mice (control *n* = 5, load *n* = 8). Data are shown as mean ± SEM. Statistical analyses were performed using repeated measures ANOVA. p <0.05 was considered significant., n.s = non-significant.


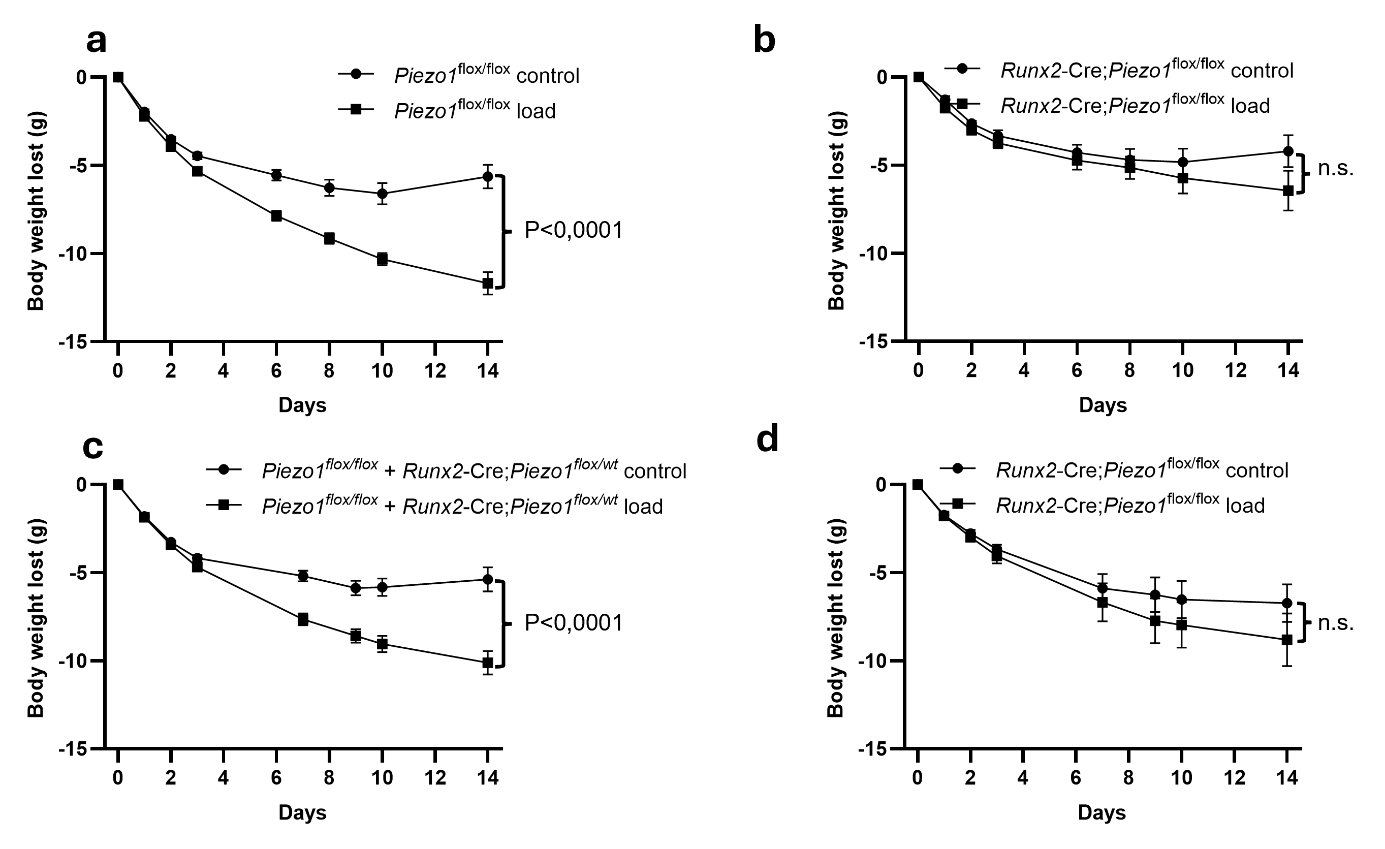


**Supplementary figure 3. Piezo1 in Runx2-expressing osteoblast-lineage cells partly mediates the effect of increased weight loading on body weight**

(A–D) The effect of increased loading on body weight presented as weight loss in grams in mice with either intact *Piezo1* expression (Piezo1^flox/flox^) or reduced *Piezo1* expression in *Runx2*-expressing osteoblast-lineage cells (*Runx2*-Cre;*Piezo1*^flox/flox^). (A) Male Piezo1^flox/flox^ mice (control *n* = 15, load *n* = 13), (B) male *Runx2*-Cre;*Piezo1*^flox/flox^ mice (control *n* = 9, load *n* = 9), (C) female Piezo1 ^flox/flox^ + Runx2-Cre;Piezo1^flox/wt^ mice (control *n* = 19, load *n* = 17), and (D) female *Runx2*-Cre;*Piezo1*^flox/flox^ mice (control *n* = 5, load *n* = 8). Data are shown as mean ± SEM. Statistical analyses were performed using repeated measures ANOVA. p <0.05 was considered significant., n.s = non-significant.

**
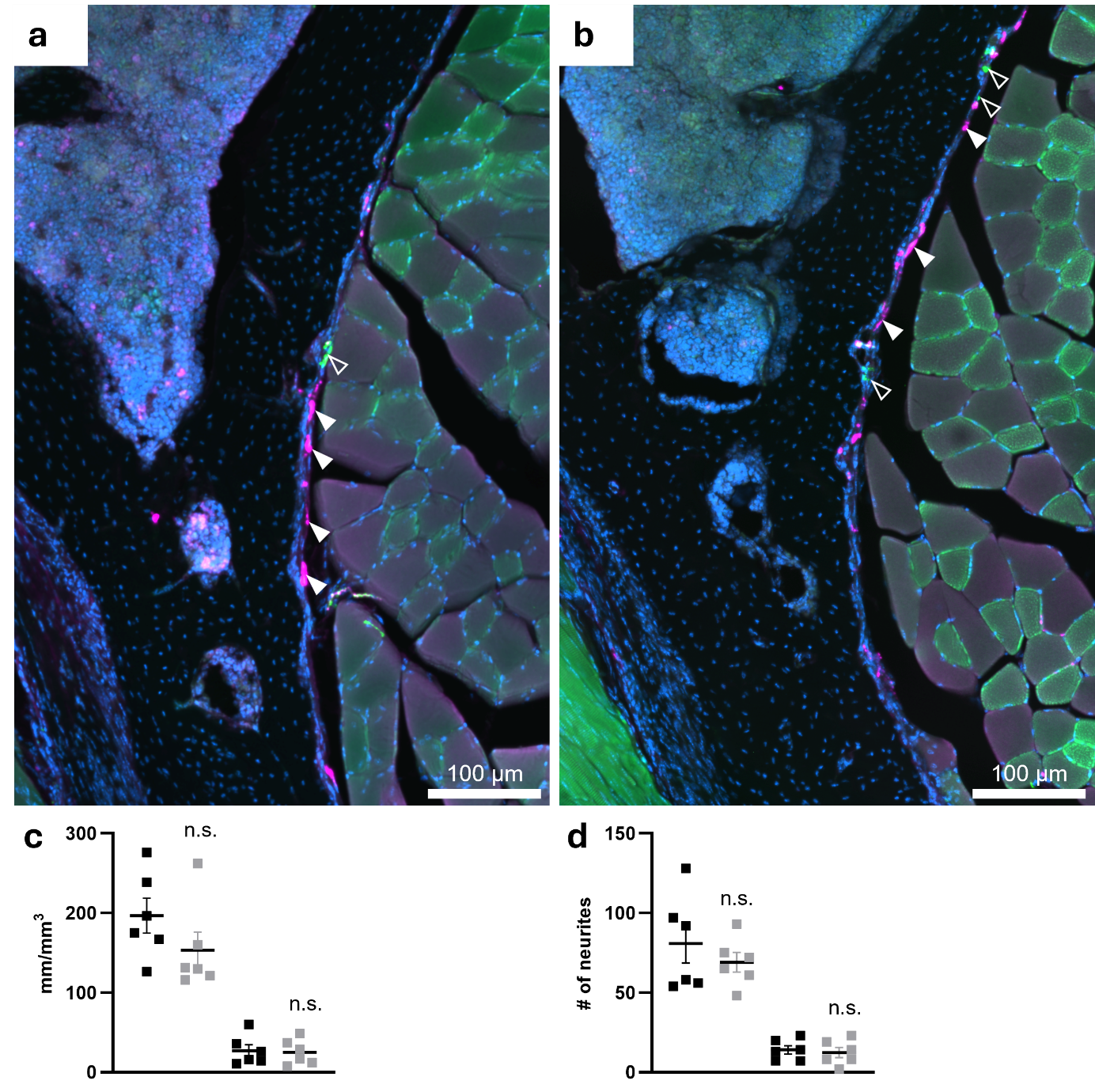
**

**Supplementary figure 4. CGRP-positive sensory nerve presence in the tibia is unaffected by increased weight loading**

After 14 days of sustained increased weight loading, tibiae from male wildtype mice were harvested and analyzed using confocal microscopy for the sensory nerve marker Calcitonin Gene-Related Peptide (CGRP; magenta, filled arrow) and the sympathetic nerve marker Tyrosine Hydroxylase (TH; green, open arrow). Representative images from **(A)** control and **(B)** load groups are shown. Quantification revealed no significant differences in **(C)** neurite length density or **(D)** neurite number between groups (*n* = 6 per group). Data are presented as mean ± SEM. Statistical analysis was performed using Student’s t test, with p<0.05 considered statistically significant.


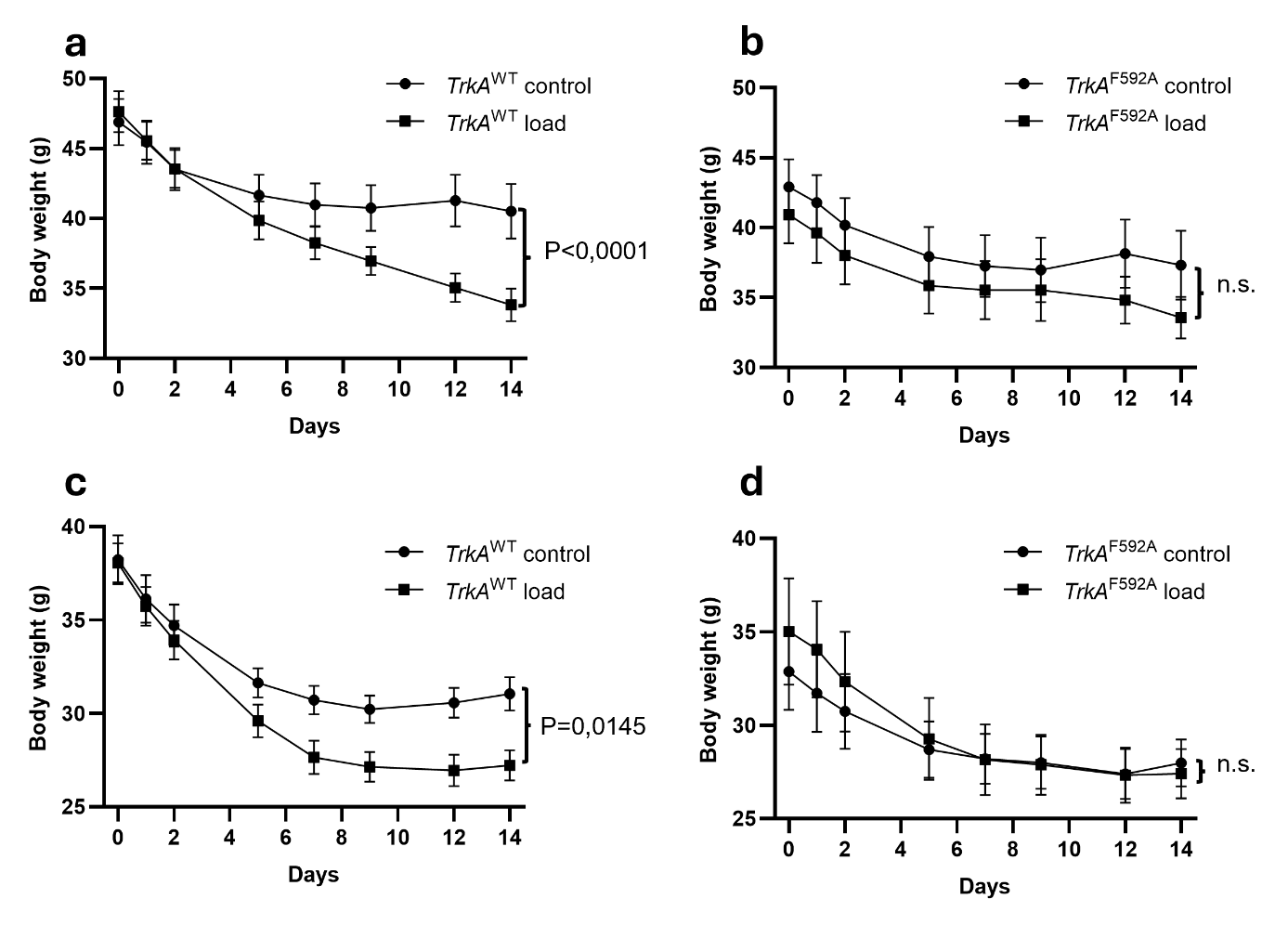


**Supplementary figure 5.** **The effect of increased weight loading on body weight is partly dependent on TrkA mediated signaling**

**(A–D**) The effect of increased loading on body weight presented as total body weight in mice with either intact peripheral nerve signaling (*TrkA^WT^*) or mice lacking functional TrkA signaling (*TrkA^F592A^*). **(A)** Male *TrkA^WT^* mice (control *n* = 7, load *n* = 7), **(B)** male *TrkA^F592A^* mice (control *n* = 7, load *n* = 6), **(C)** female *TrkA^WT^* mice (control *n* = 8, load *n* = 10), and **(D)** female *TrkA^F592A^* mice (control *n* = 7, load *n* = 5). Data are shown as mean ± SEM. Statistical analyses were performed using repeated measures ANOVA. p <0.05 was considered significant., n.s = non-significant.


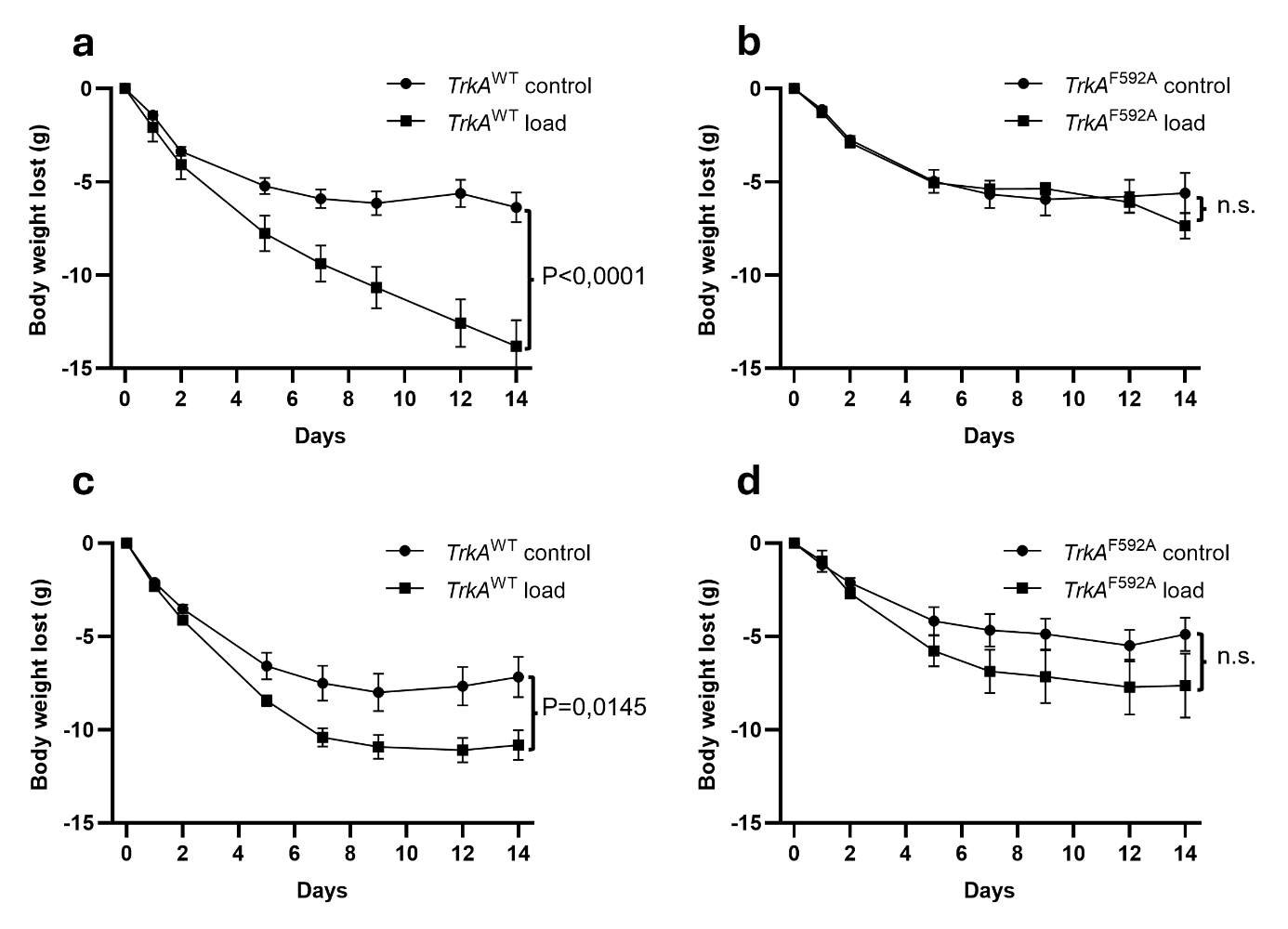


**Supplementary figure 6.** **The effect of increased weight loading on body weight is partly dependent on TrkA mediated signaling**

**(A–D**) The effect of increased loading on body weight presented as as weight loss in grams in mice with either intact peripheral nerve signaling (*TrkA^WT^*) or mice lacking functional TrkA signaling (*TrkA^F592A^*). **(A)** Male *TrkA^WT^* mice (control *n* = 7, load *n* = 7), **(B)** male *TrkA^F592A^* mice (control *n* = 7, load *n* = 6), **(C)** female *TrkA^WT^* mice (control *n* = 8, load *n* = 10), and **(D)** female *TrkA^F592A^* mice (control *n* = 7, load *n* = 5). Data are shown as mean ± SEM. Statistical analyses were performed using repeated measures ANOVA. p <0.05 was considered significant., n.s = non-significant.
